# Optimal Evolution of the Standard Genetic Code

**DOI:** 10.1007/s00239-020-09984-8

**Published:** 2021-01-24

**Authors:** Michael Yarus

**Affiliations:** Department of Molecular, Cellular and Developmental Biology, University of Colorado Boulder, Boulder, CO 80309-0347 USA

**Keywords:** Coding, Codon, Triplet, Evolve, Wobble

## Abstract

The Standard Genetic Code (SGC) exists in every known organism on Earth. SGC evolution via early unique codon assignment, then later wobble, yields coding resembling the near-universal code. Below, later wobble is shown to also create an optimal route to accurate codon assignment. Time of optimal codon assignment matches the previously defined mean time for ordered coding, exhibiting ≥ 90% of SGC order. Accurate evolution is also accessible, sufficiently frequent to appear in populations of 10^3^ to 10^4^ codes. SGC-like coding capacity, code order, and accurate assignments therefore arise together, in one attainable evolutionary intermediate. Examples, which plausibly resemble coding at evolutionary domain separation, are characterized.

## Introduction

Early evolution of the Standard Genetic Code (SGC) has been computed (Yarus 2021) by dividing code formation into time slices (passages). During a passage, with specified probability, coding triplets may either be assigned or capture mutationally related triplets for their preexisting assignment (or for related assignments) or can decay, losing assigned meaning. But also, nothing need happen during a passage. This procedure yields normal dynamic phenomena, like first- and second-order rates, as well as standard near-steady states. It is mathematically equivalent to defining typical first- and second-order kinetic constants for initiation, decay, and capture of codon assignments (Yarus 2021).

SGC-like coding tables arise by combining SGC-specific initial codon assignment (Yarus 2017, ≈ 10% randomness allowed) with coevolutionary capture (Wong 1981) that prefers amino acids with similar polar requirements (Woese et al. 1966; Mathew and Luthey-Schulten 2008). More particularly, in order to fill a coding table, wobble must arise late, for example, appearing after 20 amino acids are assigned. This leaves modern initiation and termination for a later origin, consistent with their unconserved, and thus late-arising, molecular components (Yarus 2021). Below, late wobble not only provides SGC access, but also an accessible, optimal route to SGC-like order with amino acid coding capacity.

### Simple Crick Wobble is Used

To suit primordial coding, only natural unmodified nucleotides are assumed. Thus, wobble implies only that U:G and G:U pairs are allowed at third codon nucleotide positions, as Crick first proposed (Crick 1966). Thus, XY***U*** and XY***G*** may be read either by normal base-pairing (***A*** and ***C***, respectively) or by wobble pairings (***G*** and ***U***, respectively).

### Ingredients for an Optimum

**Fig. 1:**
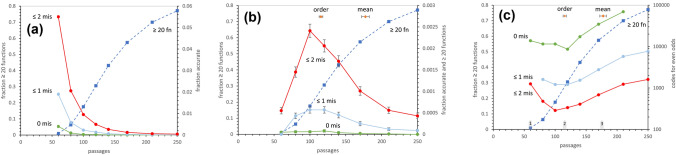
**a** Fraction of coding tables with coding capacity (≥ 20 encoded functions: left ordinate) or SGC-like assignments (0 mis, no misassignment; ≤ 1 mis, ≤ 1 misassignment; ≤ 2 mis, ≤ 2 misassignments: right ordinate), versus evolutionary time as passages. 100,000 Late wobble evolutions via Coevo_PR captures: coevolution (Wong 1981; Amirnovin 1997; Ronneberg et al. 2000) with chemical similarity (Freeland and Hurst 1998; Massey 2019) to direct amino acid choice. Probabilities per passage are *P*_mut_ = 0.04 for mutational capture, *P*_decay_ = 0.04 for assignment loss, *P*_init_ = 0.6 for initial triplet assignment, *P*_rand_ = 0.1 for random assignment (Yarus 2021). **b** Fraction of coding tables with coding capacity (≥ 20 encoded functions: left ordinate) or with both coding capacity and accurate assignment, versus evolutionary time as passages (labeling, history ,and parameters as in **a**); “order” indicates mean and s.e.m. for ≥ 20 functions with joint code order ≥ 0.9 of the SGC; “mean” indicates mean and sem for ≥ 20 encoded functions (Yarus 2021). **c** Fraction of coding tables with coding capacity (≥ 20 encoded functions: left ordinate) or number of independent codes examined to find a code with indicated accuracy half the time (right, logarithmic ordinate), versus evolutionary time as passages (labeling, history, and parameters as in **a**, **b**, save for numbered gray arrows at bottom, which mark their leftward eras for discussion)

In Fig. 1a, late-wobbling coding table evolution is shown in a fashion designed to clarify approach to an SGC-like coding table.

The fraction of coding tables encoding 20 functions or more (≥ 20 fn; on the leftward ordinate) rises with time after a lag to accumulate enough assigned triplets for 20 distinct assignments.

The fraction of coding tables correctly assigned is about an order smaller and descends with time (on the rightward ordinate), after the first near-complete tables appear around 60 passages. Eventually, Fig. 1a assignments that are 10% at random (rather than wholly SGC-like) will be made in error.

Moreover, the SGC has many wobble assignments. But an early non-wobble coding table, evolving at 60 passages, is already 67% full. Thereafter, decreasing unassigned triplets limit added wobble. Thus, as the table is filled before wobble is instituted, wobble declines and resemblance to the SGC ultimately suffers.

Three descending curves show coding accuracy: 2 or fewer assignment errors (≤ 2 mis; top line), 1 or fewer misassignments (≤ 1 mis; middle), or fully SGC-like coding with no assignment error (0 mis; bottom). In the latter case, with complete assignment precision, we touch nearest the SGC itself.

Thus, Fig. 1a shows a crucial conflict. Sufficient capacity for realistic amino acid coding increases with time; accuracy decreases. So, there is an optimum, when accuracy and coding capacity best coexist.

### An Optimum for Completion and Accuracy Defined

Figure 1b combines these coding criteria. The rising plot of capacity for ≥ 20 encoded functions is repeated to help time other events.

The other three lines, with maxima, show the fraction of codes that have both capacity to encode ≥ 20 functions and ≤ 2 assignment errors (top), ≥ 20 functions and ≤ 1 error (middle) and ≥ 20 functions with complete SGC-like codon assignment (bottom). Notably, most probable times for both qualities are similar: 100 passages (≤ 2 mis), 100–120 passages (≤ 1 mis), and 120 passages (0 mis). Optimal durations allow 39 to 44 initial triplet assignments, under these conditions (Fig. 1a, legend).

### Accessibility of SGC-Like Codes

In Fig. 1b, the abundance of competent codes is determined. This implies a code population size that must be explored to find SGC-like coding (Fig. 1c).

*P*_obs_ = probability of observation in *E*-independent evolutions, with event abundance/probability = *P*_event_:

$${\left(1-{P}_{\mathrm{event}}\right)}^{E}=\left(1-{P}_{\mathrm{obs}}\right),$$

$$E=\frac{\mathrm{ln}(1-{P}_{\mathrm{obs}})}{\mathrm{ln}(1-{P}_{\mathrm{event}})}\cong -\frac{\mathrm{ln}(1-{P}_{\mathrm{obs}})}{{P}_{\mathrm{event}}},$$

where the latter equation is accurate for somewhat rare events, *P*_event_ < ≈ 0.1. For even odds of observation, *P*_obs_ = 0.5:

$$E=\mathrm{ln}2/P_{\mathrm{event}}.$$

Abundance of accurately-formed codes, *P*_event_ (Fig. 1b), implies plausible population size *E*.

In Fig. 1c, fraction of codes specifying ≥ 20 functions again serves as time reference. Importantly, populations of about 300 (≤ 2 mis) codes, 1200 (≤ 1 mis) codes , or of 8700 (0 mis) codes would be required, at minima in Fig. 1c, to find codes that closely resemble the SGC, with even odds. SGC-like coding can therefore exist in biologically conceivable evolving populations, despite astronomically vast ensembles of possible coding schemes.

These results are consistent with previous searches (Yarus 2021), in which codes with 1 or 2 misassignments were found by searching among 600 coding tables, evolving under similar late wobble conditions. In prior searches, however, SGC-like code order was sought, rather than explicit assignment accuracy.

### A 4-Fold Optimum

**Fig. 2:**
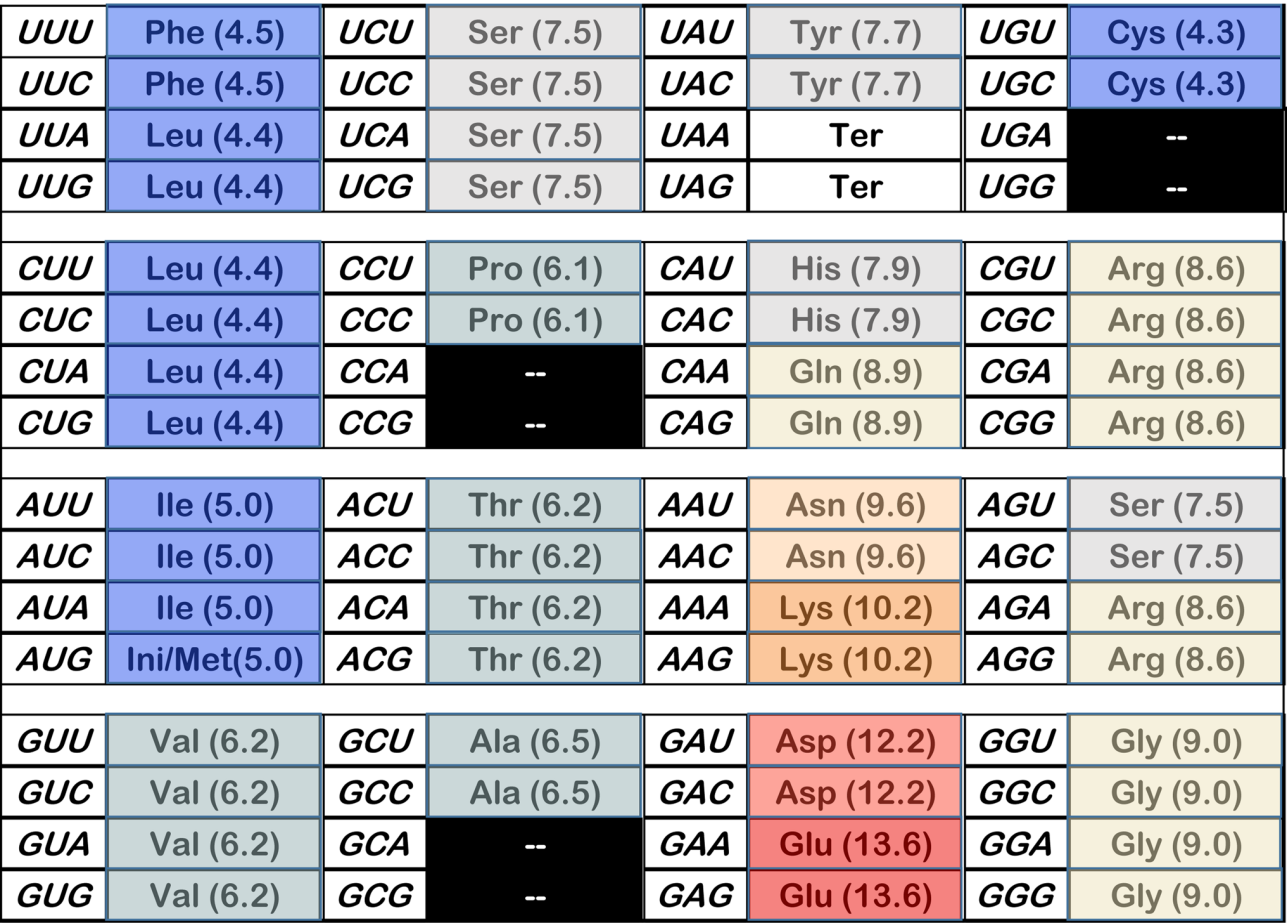
Evolved coding example from optimal time, with 20 encoded functions and no misassignments. Evolved for 115 passages using history and parameters as in Fig. 1a. More detail is in the text

Remarkably: there is a time (≈115 passages; Fig. 1b, c; Yarus 2021) and a coding state (after 42 initial assignments under these conditions; Fig. 1a) when a nascent coding table, having just adopted wobble, simultaneously possesses near-optimal spacing (identical assignments in related codons), near-optimal chemical order (related triplets associated with similar polar requirements), and SGC-like triplet sequences, the latter extending to total identity: codes with no assigned codons differing from the SGC (Figs. 1b, c, 2). Such coding tables encode 20 functions (Fig. 1a–c) and, for example, might specify all amino acids. Such competent coding precisely overlapping the SGC appears with probability ≈ 8 × 10^−5^ (Fig. 1b).

### Filling the Coding Table

To entirely resemble the SGC, one also wants full codes; most triplets assigned, but room left for late assignment of modern initiation and termination codons, and perhaps a few late, complex amino acids. In shifting to optimal time (Fig. 1b, c), unassigned triplets increase: at the 177 passage mean time (Yarus 2021), 5.4 triplets were unassigned, and at the present 115 passage optimum, 8.5 triplets are yet to be accounted for, on average. However, free triplets are also distributed. For the optimal time in Fig. 1c, 0.24 of all evolutions has 0 to 4 triplets free (using assignments for modern initiation and termination to define this hypothetical target). Because coding without misassignment has the same free distribution, this implies that codes that also have an appropriate set of unassigned triplets might be 0.24 of total, as shown in Fig. 1b, c. Using the above abundance equation—nearly full, highly ordered, 20 function, accurately assigned codes with appropriate room for known late functions appear among the following:

$$\frac{8700}{0.24}=3.6 \times {10}^{4}\,\,\mathrm{independent},\,\mathrm{late}\,\mathrm{wobbling}\,\mathrm{evolutions}.$$

If there must also be room for other late functions assigned by different means, the code population required for even odds would contain between 8.7 × 10^3^ and 3.6 × 10^4^ independent codes. Notably, the individuals required to present codes will be greater than the number of independent codes required. But this is a feasible biological ensemble; in fact, an evolving SGC might appear in a small biological group, if code-bearers are microorganisms.

### An Example Coding Table

To make numerical results tangible, consider an explicit example. Figure 2 is an evolved coding table from the likely range above. It has been colored to display amino acid chemistry as polar requirement (Woese et al. 1966; Mathew and Luthey-Schulten 2008): blue for the most hydrophobic, through light blue, then gray, beige, orange, and red, the latter for the most hydrophilic amino acids. Parenthetical numbers beside amino acid names are corrected polar requirements (Mathew and Luthey-Schulten 2008). Figure 2’s encoding was the 12,804th in a series of 115 passage evolutions, has 6 unassigned triplets, encodes 20 functions, agrees completely with SGC assignments, and has SGC-like order: compactness (spacing = 0.947; where random coding = 0.0 and SGC = 1.0), SGC-like distance (distance = 0.962), and SGC-like chemical order (dPR = 0.963).

Figure 2 visually confirms that code order and assignment accuracy coexist at 115 passages. In its calculation, 26 such inerrant coding tables were evolved. These were found among ≈ 9000 evolutions with probability 0.5, agreeing with Fig. 1b’s 8700. This implies an abundance of 7.7 × 10^−5^, again agreeing with Fig. 1b’s 8 × 10^−5^. Figure 2 varies from the SGC with gaps in canonical Pro and Ala boxes, incomplete encoding at one termination (Ter) codon and a complex, possibly late-evolving amino acid (Trp; Grosjean and Westhof 2016). Except for such arguably realistic exceptions, Fig. 2 is SGC-like.

### Time for SGC-Like Codes

Starting bloc selection (Yarus 2018) identifies early biological development as the ideal time for selection of a desired improvement. Were resemblance to the SGC selectable, such selection would work best early in coding table evolution (Fig. 1b, c). Accordingly, the SGC may be a new instance of starting bloc selection.

### Three Evolutionary Eras

Evolution under these conditions (Fig. 1a legend) can be summarized, spanning a possible starting bloc. Early events fall readily into one of three approximately equal eras. During era 1 (ends at gray arrow 1, Fig. 1c), partial coding tables are filled to produce mature coding capacity (Fig. 1a). Era 1 codes likely compete on the basis of coding capacity. However, at 60 passages and 28 initial assignments, 20 function codes still comprise < 1% of the population.

During the second, optimal era (ends at gray arrow 2, Fig. 1c), passage of about the same amount of additional time, and 13 more initial triplet assignments produce an optimum. Twenty-function late wobble coding sharply increases, 20- to 30-fold (Fig. 1b). Such coding can be highly ordered (Fig. 2): with identical assignments grouped, chemically similar amino acids associated with related triplets, and distance to the SGC short. A particularly interesting short distance exists in 7.7 × 10^−5^ of coding tables matching SGC capacity, order, and assignments simultaneously (gray arrow 2, Fig. 1c). Very SGC-like codes could be selected among 10^4^ independent codes or a few-fold more. However, if one or two differing codon assignments are tolerable for a posited selection, hundreds or thousands of codes could be sufficient to select an SGC precursor (gray arrow 2, Fig. 1c).

Figure 2 shows an evolved code example with accurate SGC-like assignments, from the end of era 2. The domains of life use similar initiation and termination triplets, but different mechanisms and molecules for interpreting them (Yarus 2021). Thus, coding for translation initiation and termination was defined before mature initiation and termination mechanisms were settled. Figure 2 therefore may parallel the genetic code near domain separation, when bacterial and archeal domains diverged.

Third era, averaging evolution, ends with 50 mean initial assignments and 20 mean era 2-encoded functions (Yarus 2021), again, after passage of another, similar, era (at gray arrow 3, Fig. 1c). It seems likely that the SGC was completed in this era, adding definitive 21st and 22nd functions, initiation and termination. But fully competent, fully ordered, fully accurate codes are ≈ 4-fold rarer at gray arrow 3 than arrow 2, defining a past optimum.

Given a credible duration for any one event, this reasoning will estimate real times on an early Earth. It does not seem overly optimistic to suppose that this will be achieved.
